# The silent burden of academia: relationships among work-related stress, chronotype, fatigue, and mindful eating

**DOI:** 10.3389/fpubh.2026.1947947

**Published:** 2026-10-30

**Authors:** Nevin Sanlier, Büşra Açıkalın Göktürk, Serra Baltacı, Zeynep Bengisu Ejder

**Affiliations:** 1 Department of Nutrition and Dietetics, Faculty of Health Sciences, Ankara Medipol University, Ankara, Türkiye; 2 Department of Gastronomy and Culinary Arts, Faculty of Fine Arts, Design and Architecture, Ankara Medipol University, Ankara, Türkiye

**Keywords:** chronotype, eating behavior, faculty member, fatigue, work stress

## Abstract

**Background:**

Academics are a professional group chronically exposed to multidimensional sources of stress, such as long working hours, publication pressure, career uncertainty, work-life imbalance, and workplace bullying. This study was conducted to examine the relationships among chronotype, occupational stress, fatigue, and mindful eating among academics.

**Method:**

The study was conducted on a total of 506 faculty members—200 men and 306 women, aged 25–65—who voluntarily agreed to participate. Participants completed a questionnaire covering demographic characteristics and dietary habits, along with the Work Stress Scale, the Morning-Evening Type Scale, the Fatigue Scale (FACIT), and the Mindful Eating Scale (MES-30).

**Results:**

It was determined that 59.5% of the academics were at a healthy body weight, while 37.1% were slightly overweight or obese. It was found that women’s work stress (21.7 ± 7.72) and fatigue (24.5 ± 8.68) scores were significantly higher than those of men (17.5 ± 6.36 and 19.6 ± 7.92, respectively) (*p* < 0.001). Men’s emotional eating scores (18.7 ± 4.08) were found to be higher than those of women (16.4 ± 4.83) (*p* < 0.001). A strong negative correlation was found between chronotype and work stress (r = −0.250, *p* < 0.001) and eating mindfulness (r = −0.409, *p* < 0.001), and a strong positive correlation with fatigue (r = 0.636, *p* < 0.001). Fatigue was identified as the strongest predictor of work stress (β = 0.537, *p* < 0.001, R^2^ = 0.432).

**Conclusion:**

This study demonstrates that fatigue is the strongest predictor of work-related stress among academics and that the negative relationship between chronotype and mindful eating indicates a marked disruption in eating behavior among individuals with an evening chronotype. The fact that female academics have significantly higher levels of work stress and fatigue than their male counterparts, and that research assistants bear the highest burden of stress and fatigue compared to those in higher-ranking positions, indicates that the academic hierarchy exacerbates psychosocial inequalities. These findings emphasize the need to prioritize the development of integrated and gender-sensitive intervention programs in academic institutions that address chronotype-based work arrangements, fatigue management, and mindful eating.

## 1 Introduction

Today, as individuals bearing significant responsibilities, academics may face certain challenges related to their working conditions and expectations. Academics are expected to help students adapt to changing life circumstances, bring them up to an adequate professional level, develop and renew themselves as individuals, solve their problems, adapt to existing conditions, continue their academic development, and withstand workplace bullying. These diverse roles place multifaceted pressures on academics (1–3). While the level of work-related stress varies across occupational groups, academics are among the groups where lifestyle and dietary changes linked to work-related stress are most frequently observed. Furthermore, it has been reported that female academics experience higher levels of work-related stress compared to their male counterparts (2). Academics’ workloads are not limited to academic duties alone (3); focusing on assigned responsibilities and delivering optimal performance requires significant attention and can ultimately lead to stress (4). It has been reported that individuals with distinctly different chronotype characteristics exhibit significant differences in numerous psychological, behavioral, and biological variables, including regular mealtimes, performance, mood, alertness, appetite, task performance, body temperature, and cortisol and melatonin secretion (5–7). One study reported that morning-type individuals demonstrate better academic performance (8). Another study noted a positive correlation between evening chronotype and academic stress scores (9). Additionally, chronotype may be associated with stress and fatigue by influencing sleep quality. It has been reported that individuals with an evening chronotype have lower sleep quality, stress, and fatigue scores (10). It has been noted that individuals with a morning chronotype demonstrate significantly higher academic performance compared to those with an evening chronotype. The evening chronotype may have a significant direct effect on sleep disorders, perceived work stressors, and the physical stress response (4). It has been found that individuals with an evening chronotype experience higher levels of work-related chronic fatigue compared to those with mixed chronotypes and morning chronotypes (5).

Chronotype can also influence an individual’s eating habits (6). When examining the relationship between chronotype, food, energy, and nutrient intake, and dietary behaviors, it is thought that evening-type individuals may have unhealthy eating habits (7). It has been reported that individuals with an evening chronotype show less adherence to a healthy diet, delayed meal timing, a tendency to skip breakfast, lower consumption of fruits and vegetables, and a greater preference for sugary foods/beverages and alcohol (11). Daily carbohydrate intake, as well as total fat, saturated fat, and sodium intake, may be higher in individuals with an evening chronotype compared to those with morning or intermediate chronotypes (12). This has been linked to an increased risk of obesity (13–15). Stress may be associated with dietary habits, eating disorders, and emotional eating behavior. Individuals exposed to stress related to work or social life are found to have a greater tendency toward hedonic hunger and emotional eating (16). It has been noted that individuals with high levels of stress and anxiety exhibit more intuitive eating behaviors (17).

Although work-related stress, chronotype, fatigue, and mindful eating have been studied individually, evidence on their combined associations among academic staff remains limited. In particular, few studies have examined whether chronotype, fatigue, and different dimensions of mindful eating are simultaneously associated with work-related stress. Therefore, this study aimed to examine these relationships and identify the factors associated with work-related stress among academic staff.

The study’s hypothesis diagram is presented in Figure 1.

**Figure 1:**
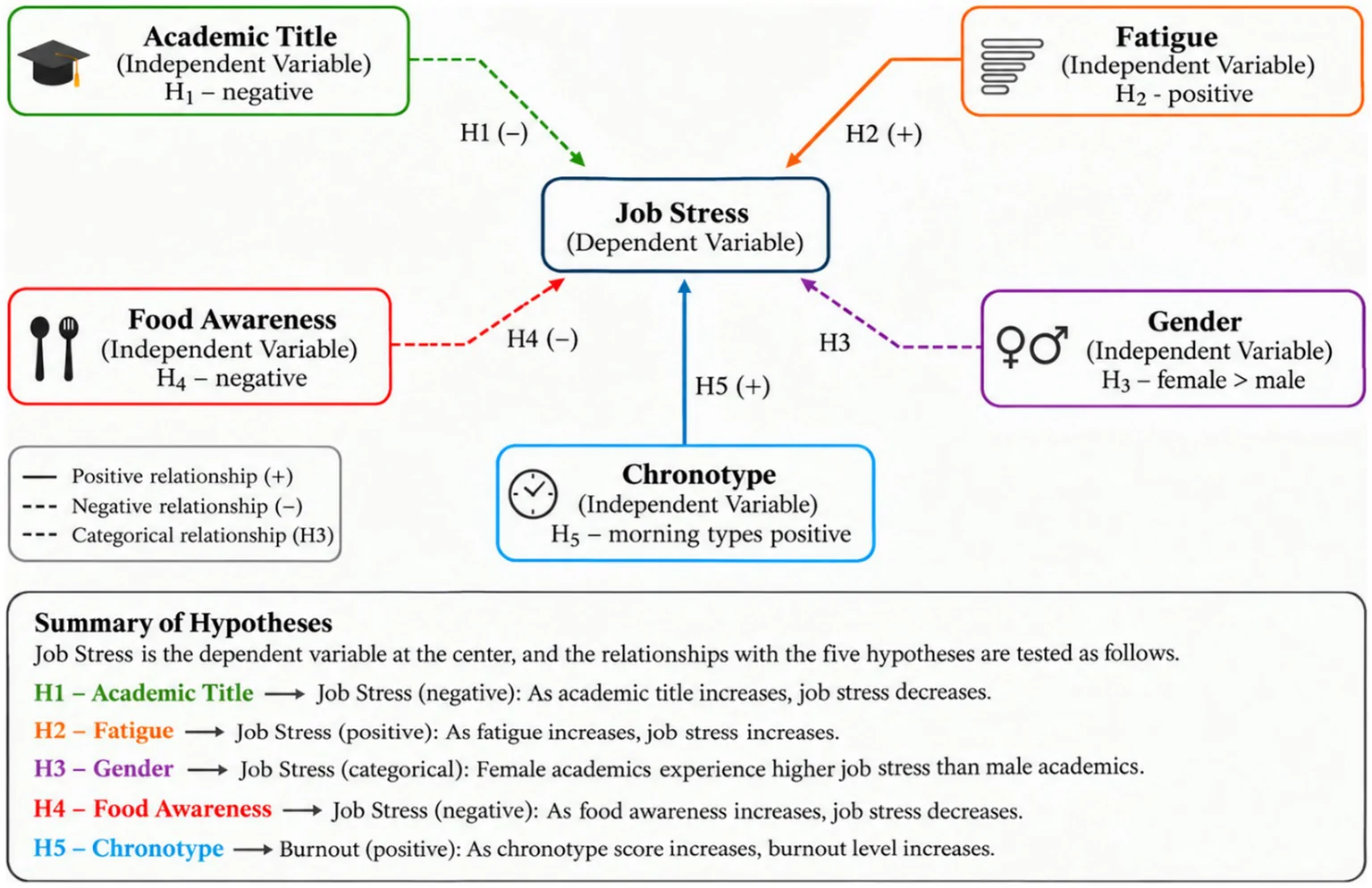
Hypothesis diagram.

The hypotheses of the study are as follows:

*Hypothesis 1*: Work-related stress decreases as academics’ academic ranks increase.

*Hypothesis 2*: As fatigue levels increase among academics, job stress Work-related stress levels increase significantly.

*Hypothesis 3*: Female academics experience higher levels of Work-related stress than male academics.

*Hypothesis 4*: As mindful eating increases, work related stress levels decrease.

*Hypothesis 5*: There is a significant positive correlation between chronotype score and fatigue levels.

## 2 Methods

This study was conducted between February 2024 and May 2025 on a total of 506 faculty members—200 men and 306 women, aged 25–65—who volunteered to participate in the research and were employed at various universities, faculties and vocational school. To determine the sample size for this study, research conducted on similar populations (academics) and using similar variables was used as a reference. An *a priori* power analysis was conducted using G*Power 3.1 to determine the minimum required sample size for the multiple linear regression analysis. Assuming a medium effect size (f^2^ = 0.15), an alpha level of 0.05, a statistical power of 90%, and three predictors, the analysis indicated that a minimum sample size of 100 participants was required. Accordingly, the final sample size was considered sufficient to detect the anticipated effect with 90% statistical power. Studies conducted on academics have shown that sample sizes typically range from approximately 150 to 250 individuals (18, 19). Based on a power analysis conducted in line with these findings, it was determined that including at least 134 individuals in the study would be sufficient; therefore, the 506 individuals included in the study were considered an adequate sample size. Participants were recruited using a convenience sampling method. Academic staff from different universities were invited to participate in the study through an online questionnaire. Participation was voluntary, and no incentives were provided.

Pregnant women, breastfeeding women, individuals under 25 or over 65 years of age, those taking medication for psychological reasons, those with chronic illnesses, and those who did not wish to participate in the study were excluded from the study. A pilot study was conducted with 40 individuals at the beginning of the study. Minor modifications were made to questions and statements that were unclear or did not function properly. The questionnaire, which included the determination of participants’ demographic characteristics, dietary habits, chronotype characteristics, and stress and fatigue levels, was administered by the researchers using a face-to-face interview technique. While collecting data face-to-face improved data quality, the potential for social desirability bias was taken into account. Approval for the study was obtained from the Ankara Medipol University Health Sciences Non-Interventional Research Ethics Committee via Decision No. 170 dated December 25, 2023.

### 2.1 Data collection and analysis

Informed consent was obtained from all participants, and the study was conducted in accordance with the principles of the Declaration of Helsinki.

The data collection tools used in the study are grouped into four eight sections:

1. Demographic information (9 questions)
2. General health information and dietary habits (6 questions)
3. Information on dietary habits (14 questions)
4. Anthropometric measurements.
5. Work Stress Scale (9 items)
6. Chronotype (Morning-Evening) Scale (19 items)
7. Fatigue Scale (13 items)
8. Mindfulness of Eating Scale-MES-30 (30 items).

### 2.2 Evaluation of anthropometric measurements

The participants’ body weight and height were measured by the researchers in accordance with standard procedures and recorded on the questionnaire. A Tanita TBF-545 portable body composition analyzer was used to measure body weight and composition. Participants’ Body Mass Index (BMI) (kg/m^2^) was calculated using the formula [Body weight (kg)/height squared (m^2^)] and evaluated according to the (20) BMI classification. Participants were categorized as having a healthy body weight (18.5 ≤ BMI ≤ 24.9 kg/m^2^), being overweight, or obese (≥25.0 kg/m^2^) (20).

### 2.3 General Work Stress Scale (GWSS)

The General Work Stress Scale was developed by De Bruin (21) and consists of 9 items rated on a 5-point Likert scale (1 = Never; 2 = Rarely; 3 = Sometimes; 4 = Most of the time; 5 = Always). The General Work-related stress Scale addresses the emotional, cognitive, motivational, and social consequences of the interaction between an individual and the perceived demands of the workplace. Scores on this scale reflect the levels of stress an individual experiences or feels, based on their own self-assessments. High scores indicate high Work-related stress while low scores indicate Work-related stress. The Cronbach’s alpha value for this study is 0.91 (21).

### 2.4 Chronotype (Morning-Evening) Questionnaire

The Chronotype (Morning-Evening) Questionnaire consists of 19 questions on a Likert scale, with four response options (Horne and Östberg, 1976); a time scale was used for questions 1, 2, and 10. Additionally, this schedule—divided into 7-h time blocks—is expressed in 15-min increments. Participants receive different scores based on the response they select for each question: for questions 3, 4, 5, 6, 7, 8, 9, 13, 14, 15, and 16, they received scores ranging from 1 to 4; for questions 1, 2, 10, 17, and 18, scores ranging from 1 to 5; for questions 11 and 19, scores ranging from 0 to 6; and for question 12, scores ranging from 0 to 5. A score range of 70–86 indicates a “definitely morning person” type; 59–69 indicates “close to a morning person”; 42–58 indicates an “intermediate type”; 31–41 indicates “close to an evening person”; and a score range of 16–30 is classified as “definitely an evening type,” resulting in five distinct circadian type classifications (). The Cronbach’s alpha value for this study is 0.812.

### 2.5 Fatigue Scale (FACIT)

The Fatigue Scale assesses self-reported fatigue and daily activities and functions. The scale consists of 13 statements that measure an individual’s fatigue over the past 7 days, and responses are scored on a scale of 0–4. It consists of a Likert-type questionnaire with response options of “none,” “very little,” “a little,” “quite a bit,” and “a lot,” and the score range is 0–52. Items 1, 2, 3, 4, 5, 6, 9, 10, 11, 12, and 13 are reverse-scored. Items 7 and 8 of the scale are scored normally. A high total score on the scale indicates a low level of fatigue in the patient, while a low total score indicates a high level of fatigue (22). The Cronbach’s alpha value for this study is 0.92.

### 2.6 Mindful Eating Scale (MES-30)

The scale developed by Framson et al. (23) consists of 30 items and is rated on a 5-point Likert scale (1: never, 2: rarely, 3: sometimes, 4: often, 5: always). The scale consists of seven subscales: disinhibition (mindless eating), emotional eating, eating control, mindfulness, eating discipline, mindful eating, and interference (influence from external factors). The scale contains 20 reverse-scored items; items 1, 7, 9, 11, 13, 15, 18, 24, 25, and 27 are scored normally, while the remaining items are reverse-scored. Reverse scoring is as follows: 1 = 5, 2 = 4, 3 = 3, 4 = 2, 5 = 1. The scale also provides a total eating awareness mindful eating score. The score is calculated as the average of the total score and the subfactor scores. A score of 3 or higher indicates high eating awareness. Mindful eating High scores on the subfactors indicate that the individual exhibits the trait associated with that subfactor in a positive manner (24). The Cronbach’s alpha value for this study is 0.733.

### 2.7 Statistical analysis

Statistical analysis of the data was performed using IBM SPSS v31. The normality of the data was assessed using the Shapiro–Wilk test and graphical methods (histogram and Q-Q plot). In this study, the descriptive statistics presented were the mean ± standard deviation or the median (minimum–maximum) for numerical variables, depending on the assumptions, and frequency (n) and percentage (%) for categorical variables.

Nonparametric methods were used for measurement values that did not follow a normal distribution. In the statistical analyses, not only *p*-values but also effect size values were considered and interpreted. In accordance with nonparametric methods, the Mann–Whitney U test (Z-table value) was used to compare measurement values between two independent groups, and the Kruskal-Wallis H test (χ^2^-table value) was used to compare measurement values among three or more independent groups. “Pearson-χ^2^” contingency tables were used to examine the relationships between two categorical variables. The Spearman correlation coefficient was used to examine the relationships between two quantitative variables that did not follow a normal distribution. Multiple linear regression analysis was performed to identify the independent variables affecting a numerical dependent variable, and the results were presented along with the coefficient of determination (R^2^). In the regression analysis, the presence of multicollinearity was assessed using VIF (Variance Inflation Factor) values. In the analyses, a *p*-value of <0.05 was considered statistically significant.

## 3 Result

This study assessed work-related stress, fatigue, chronotype, and dietary habits among a total of 506 academics—200 men and 306 women—aged 25–65. Information on the participants’ demographic characteristics is presented in Table 1.

**Table 1:** Demographic characteristics of the participants.

Variables	Male		Female		Total		χ^2^/p
	(*n* = 200)		(*n* = 306)		(*n* = 506)		
	*n*	%	*n*	%	*n*	%	
Age (year)							
25-45	127	63.5	237	77.5	364	71.9	χ^2^ = 11.661
46-65	73	36.5	69	22.5	142	28.1	*p* < 0.001
Type of faculty							
Social sciences	51	25.5	68	22.2	119	23.5	
Health sciences	45	22.5	102	33.4	147	29.1	χ^2^ = 8.40 6
Natural sciences	39	19.5	41	13.4	80	17.5	*p* = 0.078
Education sciences	40	20	57	18.6	97	19.2	
Vocatinal school							
Academic title	25	12.5	38	12.4	63	12.5	
Professor	34	17	34	11.1	68	13.4	
Associate Professor	43	21.5	53	17.3	96	19	χ^2^ = 8.832
Assistant Professor	92	46	159	52	251	49.6	*p* = 0.045
Research Assistant	31	15.5	60	19.6	91	18	
Marital status							
Single	80	40	113	36.9	193	38.1	χ^2^ = 0.484
Married	120	60	193	63.1	313	61.9	*p* = 0.87
BMI (kg/m^2^)							
Underweight	7	3.5	10	3.3	17	3.4	χ^2^ = 0.998
Healthy Weight/normal	124	62	177	57.8	301	59.5	*p* = 0.607
Overweight/obese	69	34.5	119	38.9	188	37.1	
Diagnosed illness							
Yes	22	11	62	20.3	84	16.6	χ^2^ = 7.493
No	178	89	244	79.7	422	83.4	*p* = 0.006*
Number of main meals							
1	2	1	11	3.6	13	2.6	χ^2^ = 3.641
2	81	40.5	129	42.2	210	41.5	*p* = 0.162
3	117	58.5	166	54.2	283	55.9	
Number of snacks							
None	126	63	113	36.9	239	47.2	
1	42	21	77	25.2	119	23.5	χ^2^ = 38.153
2	21	10.5	75	24.5	96	19	*p* < 0.001
3	11	5.5	41	13.4	52	10.3	

“Pearson-χ^2^” contingency tables were used to examine the relationships between two categorical variables. *p < 0.05.

60.4% of the participants were women and 39.6% were men, and 63.5% were between the ages of 25 and 45 (χ^2^ = 11.661; *p* < 0.001). It was determined that 89.0% of male academics had no diagnosed illness, while 20.3% of women had a diagnosed illness (*p* = 0.006). It was found that 30.5% of men smoke and 63.0% do not eat between-meal snacks, while 78.8% of women do not smoke and 25.2% eat one between-meal snack (*p* < 0.001).

The participants’ tea and coffee consumption and smoking habits are presented in Table 2.

**Table 2:** Individuals’ daily tea and coffee consumption habits.

Variables	Male		Female		Total		χ^2^ /p
	(*n* = 200)		(*n* = 306)		(*n* = 506)		
	*n*	%	*n*	%	*n*	%	
Daily coffee consumption							
No	63	31.5	76	24.8	139	27.5	χ^2^ = 2.695
Yes	137	68.5	230	75.2	367	72.5	*p* = 0.101
Size of daily coffee consumed							
Large (400 mL)	35	21.1	33	13.6	68	16.7	χ^2^ = 8.070
Medium (300 mL)	88	53	117	48.4	205	50.2	*p* = 0.018*
Small (200 mL)	43	25.9	92	38	135	33.1	
Size of daily Turkish coffee consumption							
1–3 cups (65–195 mL)	110	55	194	63.4	304	60.1	χ^2^ = 4.623
4–6 cups (260–390 mL)	15	7.5	23	7.5	38	7.5	*p* = 0.328
7–9 cups (455–585 mL)	6	3	5	1.6	11	2.2	
10–12 cups (650–780 mL)	1	0.5	2	0.7	3	0.6	
Does not drink any	68	34	82	26.8	150	29.6	
Type of coffee consumed							
Caffeinated	147	73.5	249	81.4	396	78.3	χ^2^ = 4.406
Decaffeinated	53	26.5	57	18.6	110	21.7	*p* = 0.036*
Daily tea consumption							
No	24	12	39	12.7	63	12.5	χ^2^ = 0.062
Yes	176	88	267	87.3	443	87.5	*p* = 0.804
Daily amount of tea consumed							
Does not drink any	16	8	37	12.1	53	10.5	χ^2^ = 21.640
1–3 tea glasses (100–300 mL)	54	27	134	43.8	188	37.2	*p* < 0.001
4–6 tea glasses (400–600 mL)	71	35.5	79	25.8	150	29.6	
7–9 tea glasses (700–900 mL)	44	22	41	13.4	85	16.8	
10–12 tea glasses (1000–1,200 mL)	15	7.5	15	4.9	30	5.9	
Smoking							
Smokes (20 cigarettes/day)	61	30.5	37	12.1	98	19.4	χ^2^ = 29.887
Does not smoke	115	57.5	241	78.8	356	70.4	*p* < 0.001
Has quit	24	12	28	9.1	52	10.2	

“Pearson-χ^2^” contingency tables were used to examine the relationships between two categorical variables. *p < 0.05.

It was found that 21.1% of men consumed large-sized coffee, 26.5% consumed decaffeinated coffee, and 81.4% consumed caffeinated coffee; 48.4% of women consumed medium-sized coffee, 18.6% consumed decaffeinated coffee, and 81.4% consumed caffeinated coffee (coffee size χ^2^ = 8.070, *p* = 0.018; coffee type χ^2^ = 4.406, *p* = 0.036). Academics generally prefer caffeinated coffee (78.3%). It was determined that 35.5% of men consume 4–6 tea cups, while 43.8% of women consume 1–3 tea cups of tea (χ^2^ = 21.640; *p* < 0.001).

The relationship between academic rank, coffee consumption, and scale scores is shown in Table 3.

**Table 3:** Scores on scales measuring Work-related stress chronotype, fatigue, and mindful eating in relation to academic rank and coffee consumption.

Variable	*n*	Work stress	Chronotype	Fatigue	Mindful eating
		$\bar{X}\pm \mathrm{SS}$	$\bar{X}\pm \mathrm{SS}$	$\bar{X}\pm \mathrm{SS}$	$\bar{X}\pm \mathrm{SS}$
Academic title					
Professor ^(1)^	68	18.1 ± 6.98	45.5 ± 8.73	21.4 ± 9.48	103.2 ± 13.69
Associate Professor ^(2)^	96	19.3 ± 7.41	45.0 ± 9.09	22.4 ± 8.18	100.4 ± 14.09
Assistant Professor ^(3)^	137	19.7 ± 7.45	43.7 ± 7.93	21.8 ± 8.40	99.4 ± 13.50
Lecturer ^(4)^	114	19.2 ± 6.52	41.6 ± 9.38	21.7 ± 8.90	98.8 ± 16.02
Research Assistant ^(5)^	91	23.9 ± 7.96	43.7 ± 8.16	25.8 ± 8.32	95.7 ± 14.17
χ^2^/p		χ^2^ = 28.893	χ^2^ = 12.645	χ^2^ = 15.647	χ^2^ = 11.473
		*p* < 0.001	*p* = 0.013*	*p* = 0.004*	*p* = 0.022*

For data that do not follow a normal distribution, the “Mann–Whitney U” test (Z-table value) was used to compare the measurement values of two independent groups, and the “Kruskal-Wallis H” test (χ^2^-table value) was used to compare three or more independent groups. *p < 0.05.

General work stress among research assistants (23.9 ± 7.96) was higher than that of other academics (χ2 = 28.893, *p* < 0.001), and their chronotype scores were lower than those of professors (45.5 ± 8.73) and associate professors (45.0 ± 9.09) compared to research assistants (41.6 ± 9.38), (χ^2^ = 12.645, *p* = 0.013), and that the fatigue levels of research assistants (25.8 ± 8.32) were lower than those of professors (21.4 ± 9.48), associate professors (22.4 ± 8.18), and assistant professors (21.8 ± 8.40) (χ^2^ = 15.647, *p* = 0.004), and that the eating awareness mindful eating of professors (103.2 ± 13.69) and associate professors (100.4 ± 14.09) was found to be significantly higher than that of research assistants (95.7 ± 14.17) (χ^2^ = 11.473, *p* = 0.022).

Table 4 shows the relationship between individuals’ scale scores and their age, marital status, BMI, and the type of faculty where they work.

**Table 4:** Comparison of total scores on the work stress, chronotype, fatigue, and mindful eating scales by age, marital status, BMI, college, and coffee consumption.

Variables	n	Work Stress	Chronotype	Fatigue	Mindful eating
		$\bar{X}\pm \mathrm{SS}$	$\bar{X}\pm \mathrm{SS}$	$\bar{X}\pm \mathrm{SS}$	$\bar{X}\pm \mathrm{SS}$
Age (year)					
25–45	364	21.0 ± 7.52	42.9 ± 8.42	23.2 ± 8.52	98.1 ± 14.20
46–65	142	17.5 ± 6.79	45.9 ± 8.86	20.9 ± 9.02	102.5 ± 14.71
Z/p		Z = −4.982	Z = −3.357	Z = −2.693	Z = −2.983
		*p* < 0.001	p < 0.001	*p* = 0.007*	*p* = 0.003*
Marital status					
Single	193	21.19 ± 7.12	42.2 ± 8.20	23.6 ± 8.45	97.2 ± 13.74
Married	313	19.33 ± 7.63	44.8 ± 7.88	21.9 ± 8.83	100.6 ± 14.76
Z/p		Z = −3.206	Z = −3.506	Z = −2.225	Z = −2.821
		*p* = 0.001	*p* < 0.001	*p* = 0.026*	*p* = 0.005*
BMI (kg/m^2^)					
Underweight	17	22.94 ± 9.26	40.9 ± 11.75	24.6 ± 9.36	96.5 ± 18.45
Healthy weight /Normal	301	20.00 ± 7.31	43.7 ± 8.82	22.4 ± 8.45	99.3 ± 14.28
Overweight/obese	188	9.83 ± 7.58	44.3 ± 8.02	22.6 ± 9.09	99.6 ± 14.07
χ^2^/p		χ^2^ = 1.376	χ^2^ = 1.555	χ^2^ = 1.078	χ^2^ = 0.587
		*p* = 0.502	*p* = 0.460	*p* = 0.583	*p* = 0.745
Type of faculty					
Social ^(1)^	119	20.90 ± 8.08	42.9 ± 9.65	23.6 ± 8.79	98.1 ± 14.85
Health ^(2)^	147	20.52 ± 6.44	43.3 ± 8.15	21.4 ± 8.65	98.3 ± 13.67
Natural ^(3)^	80	19.51 ± 7.26	45.1 ± 9.08	21.3 ± 8.74	101.3 ± 13.41
Education ^(4)^	97	18.51 ± 7.67	45.5 ± 7.85	20.8 ± 8.45	102.2 ± 15.24
Vocational School ^(5)^	63	20.30 ± 6.17	42.7 ± 8.03	25.1 ± 8.50	97.0 ± 15.03
χ^2^/p		χ^2^ = 8.107	χ^2^ = 9.111	χ^2^ = 14.844	χ^2^ = 9.905
		*p* = 0.088	*p* = 0.058	*p* = 0.005*	*p* = 0.042*
Coffee consumption No 139		21.1 ± 7.42	44.9 ± 7.77	23.6 ± 8.96	97.2 ± 14.19
Yes 367		19.6 ± 7.48	43.4 ± 8.93	22.2 ± 8.60	100.1 ± 14.50
Z/p		Z = −2.179	Z = −1.292	Z = −1.760	Z = −2.447
		*p* = 0.029*	*p* = 0.196	*p* = 0.078	*p* = 0.014*

For data that do not follow a normal distribution, the “Mann–Whitney U” test (Z-table value) was used to compare the measurement values of two independent groups, and the “Kruskal-Wallis H” test (χ^2^-table value) was used to compare three or more independent groups. *p < 0.05.

It was found that work-related stress and fatigue among academics aged 25–45 were higher than those among those aged 46–65; chronotype and mindful eating among those aged 46–65 were higher than those among those aged 25–45; work-related stress and fatigue among single individuals were higher than those among married individuals; and married individuals’ chronotype and eating awareness mindful eating were found to be higher than those of single individuals (*p* < 0.05). The scale scores for work-related stress, chronotype, fatigue, and eating awareness mindful eating among faculty members working in the faculties of health, science, and education, as well as at the Community College (MYO), were determined to be 20.52 ± 6.44, 43.3 ± 8.15, 21.4 ± 8.65, and 98.3 ± 13.67, respectively. It was determined that fatigue levels among faculty members working at community colleges were significantly higher than those in the health, science, and education faculties (*p* < 0.05). It was found that daily coffee consumption among faculty members was associated with Work-related stress (Z = −2.179, *p* = 0.029) and mindful eating (Z = −2.447, *p* = 0.014). It was also found that mindful eating among coffee drinkers was significantly higher than among non-coffee drinkers.

The relationship between participants’ scale scores and gender is presented in Table 5.

**Table 5:** Scores for work stress, chronotype, fatigue, and mindful eating among academics, by gender.

Variables	Male (*n* = 200)	Female (*n* = 306)	Total (*n* = 506)	Z/p
	$\bar{X}\pm \mathrm{SS}$	$\bar{X}\pm \mathrm{SS}$	$\bar{X}\pm \mathrm{SS}$	
Work stress	17.5 ± 6.36	21.7 ± 7.72	20.0 ± 7.49	Z = −5.954 *p* < 0.001
Chronotype	44.8 ± 7.95	43.6 ± 9.03	43.8 ± 8.64	Z = −2.065 *p* = 0.039*
Fatigue	19.6 ± 7.92	24.5 ± 8.68	22.6 ± 8.71	Z = −6.084 *p* < 0.001
Mindful eating	99.1 ± 13.08	99.4 ± 15.32	99.3 ± 14.46	Z = −0.017 *p* = 0.986
Disinhibasyon	16.7 ± 3.78	16.7 ± 4.05	16.7 ± 3.94	Z = −0.006 *p* = 0.996
Emotional eating	18.7 ± 4.08	16.4 ± 4.83	17.3 ± 4.67	Z = −5.326 *p* < 0.001
Eating control	20.3 ± 4.11	21.4 ± 4.75	20.9 ± 4.54	Z = −2.637 *p* = 0.008*
Awareness	15.97 ± 2.02	15.7 ± 1.99	15.8 ± 2.01	Z = −0.977 *p* = 0.328
Eating Discipline	12.1 ± 2.96	13.3 ± 2.79	12.8 ± 2.91	Z = −4.191 *p* < 0.001
Conscious eating	16.1 ± 2.82	16.2 ± 2.81	16.2 ± 2.81	Z = −0.138 *p* = 0.891
Interference	7.1 ± 1.62	6.9 ± 1.76	7.0 ± 1.71	Z = −0.742 *p* = 0.458

For data that do not follow a normal distribution, the “Mann–Whitney U” test (Z-table value) was used to compare the measurement values of two independent groups. ^*^p < 0.05.

The mean scale scores for work-related stress, chronotype, fatigue, and mindful eating among men were 17.5 ± 6.36, 44.8 ± 7.95, 19.6 ± 7.92, and 99.1 ± 13.08, respectively, while for women they were 21.7 ± 7.72, 43.6 ± 9.03, 24.5 ± 8.68, and 99.4 ± 15.32. Significant differences were found in work-related stress (*p* < 0.001), chronotype (*p* = 0.039), fatigue (*p* < 0.001), emotional eating (*p* < 0.001), eating control (*p* < 0.05), and eating discipline (*p* < 0.001) scores by gender. Women’s scores for work-related stress (21.7 ± 7.72), fatigue (24.5 ± 8.68), eating control (21.4 ± 4.75), and eating discipline (13.3 ± 2.79) were higher than those of men (17.5 ± 6.36, 19.6 ± 7.92, 20.3 ± 4.11, 12.1 ± 2.96, respectively), while men’s chronotype (44.8 ± 7.95) and emotional eating (18.7 ± 4.08) scores were found to be higher than those of women (43.6 ± 9.03, 16.4 ± 4.83, respectively) (*p* < 0.05).

A heat map illustrating the relationships between work-related stress and other measurement tools is presented in Figure 2.

**Figure 2:**
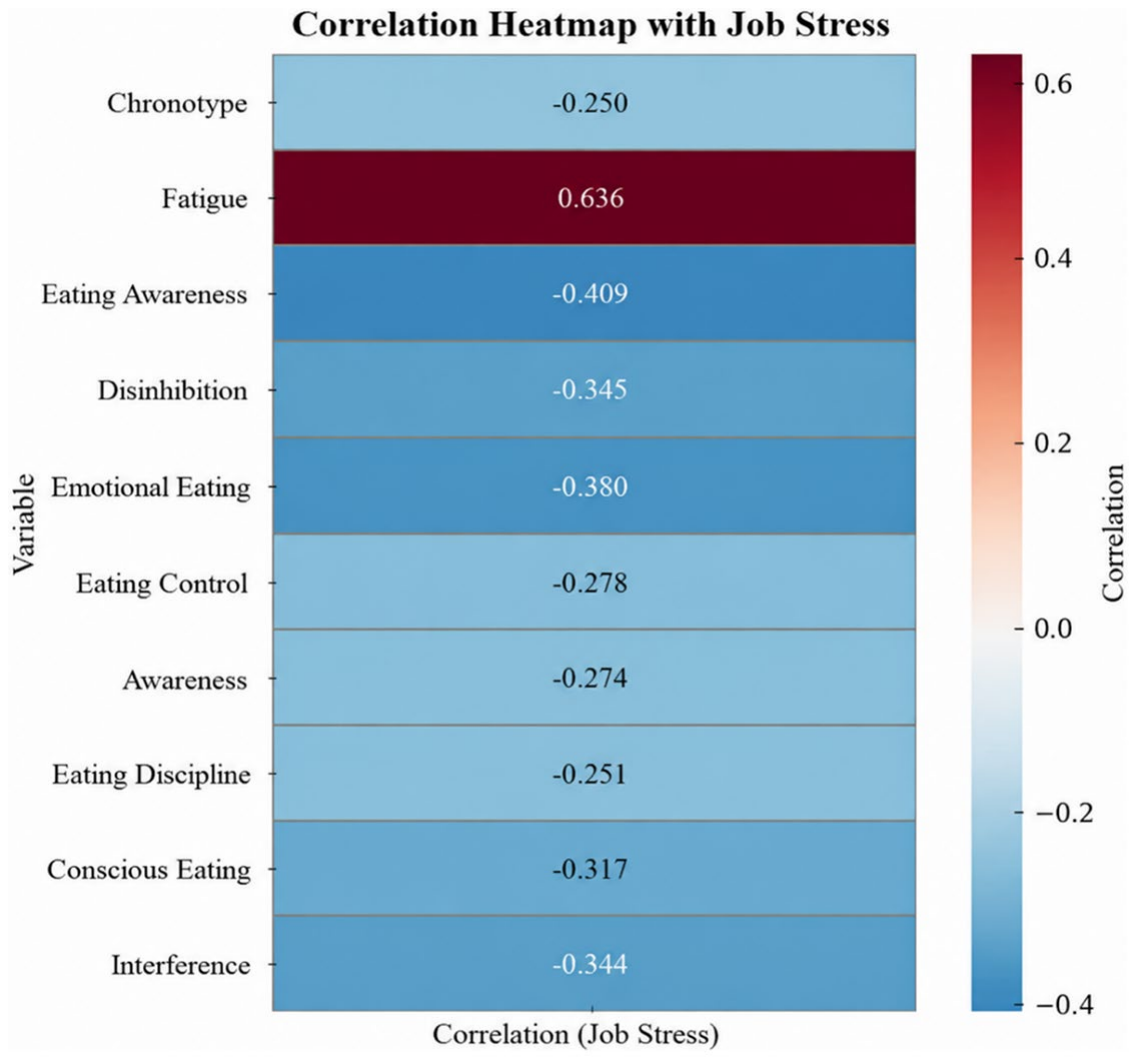
Heat map of the relationships between work stress and other measurement tools—heat map.

Negative correlations were found between chronotype and work stress, mindful eating, disinhibition, emotional eating, eating control, mindfulness, eating discipline, mindful eating, and interference, while a positive correlation was found with fatigue. As the chronotype score increases, the scale scores for work stress and eating awareness mindful eating decrease, while the fatigue score increases. Negative correlations were found between work stress and chronotype (r = −0.250), fatigue (r = 0.636), and mindful eating awareness (r = −0.409), disinhibition, emotional eating, eating control, mindfulness, eating discipline, mindful eating, and interference (*p* < 0.001). As chronotype, mindful eating, disinhibition, emotional eating, eating control, mindfulness, eating discipline, mindful eating, and interference increased, work-related stress decreased (*p* < 0.001).

The regression analysis of the factors affecting general work stress is presented in Table 6.

**Table 6:** Multiple linear regression analysis of other parameters affecting work-related stress.

Variables	B	S.H	β	t	*p*	%95C.I.	
						Lower	Upper
Constant	24.474	3.085		7.933	<0.001	18.412	30.536
Chronotype	−0.028	0.033	−0.032	−0.844	0.399	−0.092	0.037
Fatigue	0.462	0.035	0.537	13.129	<0.001	0.393	0.531
Disinhibition	0.005	0.120	0.002	0.038	0.970	−0.231	0.241
Emotional eating	−0.061	0.079	−0.038	−0.773	0.440	−0.217	0.095
Eating control	0.129	0.092	0.078	1.405	0.161	−0.051	0.309
Awareness	−0.478	0.133	−0.128	−3.587	<0.001	−0.740	−0.216
Eating discipline	−0.080	0.095	−0.031	−0.843	0.400	−0.266	0.106
Conscious eating	−0.283	0.122	−0.106	−2.316	0.021*	−0.522	−0.043
Interference	−0.312	0.207	−0.071	−1.507	0.132	−0.720	0.095
R = 0.657	R^2^ = 0.432	*F* = 41.919	*p* < 0.001				

^*^p < 0.05.

Multiple linear regression analysis was used to examine the independent associations of chronotype, fatigue, and mindful eating dimensions with work-related stress when these variables were considered simultaneously. The regression model as a whole is statistically significant (*F* = 41.919; *p* < 0.001). It was determined that the effects of fatigue, mindfulness, and mindful eating on work-related stress are statistically significant (*p* < 0.05). It was determined that as fatigue increases, work stress increases, and as mindfulness and mindful eating increase, work stress decreases. Fatigue was identified as the factor with the greatest effect on work stress (the independent variables explained 43.2% of the variation in the dependent variables; R^2^ = 0.432).

The findings regarding the assessment of regression assumptions are presented in Table 7. In the assessment of regression assumptions, a Durbin–Watson statistic of 1.829 indicated that there was no issue regarding the independence of the residuals. HC3 robust standard errors were used to reduce the potential influence of heteroskedasticity on the estimated standard errors. The fact that the VIF values ranged from 1.11 to 3.49 and the tolerance values from 0.287 to 0.899 indicated that there was no significant multicollinearity among the variables. The Ramsey RESET test also did not reveal any significant issues regarding model specification (*p* = 0.123). However, a statistically significant deviation from a normal distribution was detected in the residuals (Shapiro–Wilk *p* < 0.001). Given that normality tests exhibit high sensitivity to small deviations in large samples and considering the large sample size of this study (n = 506), it was determined that this deviation would not significantly affect the regression estimates. Furthermore, since heteroscedasticity was detected using the Breusch–Pagan test (*p* = 0.001), HC3 robust standard errors—which are resistant to heteroscedasticity—were used in statistical inferences. Even after adjusting for HC3, the results remained unchanged; fatigue was positively associated with work stress (B = 0.462; β = 0.537; *p* < 0.001), while mindfulness (B = -0.478; β = −0.128; *p* = 0.001), and mindful eating (B = -0.283; β = −0.106; *p* = 0.018) were negatively and independently associated with work stress. These findings demonstrate that the core results of the regression analysis remain valid even after a robust analysis for heteroscedasticity, indicating that the results are reliable.

**Table 7:** Assessment of regression assumptions.

Variables	B	β	Classic p	HC3 Robust SH	HC3 t	HC3 p	Robust %95 GA
Chronotype	−0.028	−0.032	0.399	0.040	−0.681	0.496	−0.107–0.052
Fatigue	**0.462**	**0.537**	<0.001	**0.040**	**11.596**	**<0.001**	**0.383–0.540**
Disinhibition	0.005	0.002	0.970	0.113	0.040	0.968	−0.217–0.226
Emotional eating	−0.061	−0.038	0.440	0.082	−0.745	0.457	−0.223–0.101
Eating control	0.129	0,078	0,161	0,092	1,405	0,161	−0.051–0.309
Awareness	**−0.478**	**−0.128**	<0.001	**0.150**	**−3.198**	**0.001**	**−0.772–−0.184**
Eating discipline	−0.080	−0.031	0.400	0.103	−0.779	0.437	−0.282–0.122
Conscious eating	**−0.283**	−**0.106**	0,021	**0,119**	**−2,371**	**0,018**	**−0.517–0.048**
Interference	−0.312	−0.071	0.132	0.214	−1.462	0.144	−0.732–0.107

Bold values indicate statistically significant results (*p* < 0.05) based on HC3 robust standard errors.

## 4 Discussion

Due to intense working conditions with no set working hours, academics constitute a vulnerable professional group in terms of work-related stress, fatigue, and chronotype differences. Multidimensional sources of stress—such as teaching, research, administrative responsibilities, workplace bullying, and administrative pressure—adversely affect academics’ mental health; it has been reported that the university environment jeopardizes both physical and psychological well-being (24). This situation sets the stage for occupational stress and burnout among the academic workforce, seriously threatening long-term sustainability (25, 26). While career development stands out as the most prominent source of stress among academics (27), the tendency toward long working hours also negatively disrupts work-life balance (28).

This study found that women’s scores on food control and dietary discipline were significantly higher than men’s, while men’s scores on emotional eating were significantly higher than women’s. These findings suggest that eating behaviors may be shaped differently by biological, psychological, and sociocultural factors depending on gender. The high food control and discipline scores observed in women can be interpreted as a reflection of the social pressures women face regarding body weight, physical appearance, and adherence to healthy eating norms. In contrast, the high emotional eating scores observed in men may indicate that food serves as a regulatory tool for coping with stress, anger, or negative emotional states. This pattern may become more pronounced in societies where traditional gender roles restrict emotional expression; such restrictions may lead men to turn to eating behaviors as a means of regulating their emotions. When evaluated as a whole, the findings underscore the importance of considering gender as a fundamental variable in the assessment and interpretation of eating behaviors.

One study reported that men tend to skip meals, while women consume main and snack meals more regularly (29). Another study noted that women tend to have habits such as preferring to eat more meals throughout the day than men, while men are more likely to skip breakfast (30). There are studies reporting that women consume more coffee and tea than men (31, 32); in addition, it has been reported that women’s daily caffeine intake is higher than that of men (33). In line with these findings, the present study also found that women consumed more coffee, while men consumed more tea (Table 2). In both genders, consumption of caffeinated coffee was higher than that of decaffeinated coffee, and total caffeine intake among women significantly exceeded that of men. When this finding is evaluated in conjunction with studies reporting a negative correlation between caffeine intake and chronotype and emotional eating scores (34), it indicates that caffeine consumption must be considered not only in its physiological aspects but also in its psychological and behavioral dimensions. It was found that work stress scores were significantly higher among non-coffee drinkers compared to coffee drinkers, while coffee drinkers’ mindful eating scores were significantly higher compared to non-coffee drinkers (Table 3). These findings suggest that coffee consumption reflects not merely a beverage preference but may also be a component of a broader pattern related to lifestyle, stress management, and eating behaviors. However, it should be noted that the effects of caffeine consumption may vary depending on individual tolerance, dose, and timing of consumption, and it is recommended not to exceed a daily caffeine intake limit of 400 mg (35).

It has been reported that stressful working conditions among academics may trigger emotional eating through work-related stress and fatigue, thereby paving the way for unhealthy eating patterns (36). It is believed that the strong link between fatigue and work-related stress—resulting from physical and mental exhaustion—may lead to an increase in unhealthy food choices and a reduction in control over eating behavior. It has been reported that women experience higher levels of emotional work stress compared to men in the workplace (37), and that their work stress scores are also significantly higher than those of men (38). It has been reported that anxiety levels among female academics also exceed those of men alongside work-related stress (39), and it is assessed that this difference may be related to the burden arising from the simultaneous fulfillment of work and family roles, in addition to gender inequality in the workplace. Findings from the current study, however, indicate that while Work-related stress decreases with age, the workload increases. This pattern is thought to stem from increased job control among senior faculty members, alongside the development of professional experience and coping strategies. Indeed, it is assessed that the capacity to cope with stress also develops relatively as academic rank increases.

It has been reported that work-related stress may restrict healthy food choices, thereby increasing emotional eating behaviors and leading to a significant decline in dietary quality (40). Indeed, it has been reported that emotional eating behaviors related to work stress are prevalent among academics, and that men have higher scores for emotional eating (41) and uncontrolled eating compared to women (42). Chronotype, another important variable influencing eating behaviors, also differs by gender; it has been suggested that this difference may play a decisive role in mindful eating and eating behaviors (43). In this regard, it has been found that male academics have both higher chronotype scores (morning-type orientation) and higher levels of uncontrolled eating behavior compared to women (6).

Consistent with the literature, the present study also found that women’s scores on work stress, fatigue, eating control, and eating discipline were significantly higher than men’s, while men’s scores on chronotype and emotional eating were significantly higher than women’s (*p* < 0.05, Table 6). According to the findings, while women experience higher levels of stress and are more mindful of their eating than men, men have higher levels of fatigue and are more prone to emotional eating.

Academics are considered a professional group at risk for high Work-related stress and fatigue, poor sleep quality, and negative eating behaviors—such as uncontrolled and emotional eating—due to their intense work pace (44). This situation aligns with our findings, given that having an evening-type chronotype does not imply lower levels of mindful eating (or, conversely, higher levels of uncontrolled eating). When examined in terms of chronotype, it has been reported that as the “morning-type” orientation strengthens, work stress and emotional eating scores decrease (45); conversely, academics with an “evening-type” chronotype are more likely to experience a lack of control over their eating behavior and have higher emotional eating scores (46). Similarly, it has been reported that academic stress scores are higher among individuals with an evening chronotype (47). When examining the relationship between work stress, fatigue, and eating behaviors, it is suggested that individuals experiencing burnout exhibit unhealthy eating patterns, such as mindless and uncontrolled eating (9). It has been emphasized that a decrease in mindful eating and an increase in unhealthy eating behaviors are positively associated with workload, work stress, and fatigue (40); in a study conducted in Japan, it was found that as work stress and fatigue levels increased, the tendency toward fast eating and emotional eating became stronger, and that this effect was more pronounced in men than in women (48). However, there are also studies in the relevant literature that have failed to identify a significant relationship between chronotype and work stressors or stress levels (49). The findings from the present study are consistent with the general literature: as work stress increases, scores for mindful eating, emotional eating, eating control, eating discipline, conscious nutrition, and chronotype (evening-oriented tendency) decrease; meanwhile, fatigue levels rise alongside work stress (Figure 1). Furthermore, it has been reported that young academics have higher levels of work-related stress and relatively more limited stress management skills (50); this finding once again supports the decisive role of professional experience in stress management.

It has been reported that Work-related stress levels tend to decrease with increasing age and seniority (51), while among academics, both job stress Work-related stress and fatigue levels decline as age and academic rank increase (52). Consistent with these findings, the present study found that research assistants had significantly higher job stress Work-related stress and fatigue scores compared to professors, associate professors, and assistant professors, while professors and associate professors had higher chronotype and mindful eating scores compared to research assistants (Table 3). It is believed that this pattern stems from increased autonomy over work among senior academics, which develops alongside professional experience and coping strategies.

While some studies indicate that married individuals exhibit lower job stress Work-related stress and fatigue, yet higher job satisfaction and well-being, compared to single individuals (53), there is also a study reporting that married individuals experience higher occupational stress (54). This inconsistency may be attributed to differences in sample characteristics and sociocultural context.

In terms of chronotype characteristics, it has been reported that the “morning person” tendency strengthens with age, leading to an increased tendency to go to bed early and wake up early, while the “evening person” chronotype is observed more frequently in men than in women (55). It has also been found that chronotype scores increase with age (56) and that the duration of nighttime wakefulness decreases among academics (57). It has been suggested that unhealthy eating patterns are more common among young and single individuals (58). Indeed, it has been found that young adults develop more negative eating behaviors compared to middle-aged adults (59), and that single individuals have higher scores for skipping meals, mindless eating, and emotional eating—and lower scores for healthy eating competence—compared to married individuals (60).

It is well known that high levels of work-related stress negatively affect individuals’ food intake and eating patterns, and are associated with various chronic diseases, particularly obesity. In this context, it has been suggested that the combination of unhealthy eating behaviors, chronic stress, and a night-oriented eating pattern (evening chronotype) may be associated with an increased risk of BMI and obesity. Indeed, it has been reported that the evening chronotype is associated with a higher BMI in women (61) and that evening-oriented individuals are more prone to obesity linked to unhealthy eating habits (11). It has also been reported that an increase in emotional eating scores and a decrease in eating discipline are associated with a rise in BMI (62); among academics, an evening chronotype has been linked to higher BMI values (63). On the other hand, meal planning structured according to chronotype has also been found to play a decisive role in BMI; concentrating the majority of daily total energy intake in the morning hours has been associated with a lower likelihood of obesity in individuals with a morning chronotype (64). However, the present study did not find a significant association between BMI and work stress, chronotype, fatigue, and mindful eating.

In the present study, statistically significant differences were found in work stress, chronotype, fatigue, and mindful eating scores across age groups (*p* < 0.001, Table 4). This finding suggests that age may play a decisive role in shaping academic lifestyle and individual behavioral patterns. It is believed that academics in the early stages of their careers may experience greater stress and fatigue due to heavy workloads and high performance expectations, whereas in older age groups, increased professional experience and developed coping mechanisms may help balance this burden. Age-related changes in biological rhythms can also be considered among the factors that partially explain the differences in chronotype and mindful eating scores. Furthermore, it was found that single academics had significantly higher work stress and fatigue scores compared to married academics, while married academics had significantly higher chronotype and mindful eating scores compared to single academics (Table 4). It has been reported that social support mechanisms enhance psychological resilience and mitigate the adverse effects of stress (65). It is believed that the social and emotional support married individuals receive from their spouses can reduce their perception of stress by facilitating coping with work-related stressors.

### 4.1 Limitations

The findings of this study should be interpreted within the context of certain limitations. First, due to its cross-sectional design, the relationships between chronotype, work stress, fatigue, and eating behaviors cannot be interpreted in terms of causality. Furthermore, the fact that the data were collected via self-report methods may create a basis for measurement errors such as recall bias and social desirability bias. Furthermore, the fact that the sample consists solely of academics limits the generalizability of the findings to other occupational groups. Finally, the lack of comprehensive control for potential confounding variables—such as sleep quality, physical activity levels, and detailed dietary intake—partially complicates the interpretation of the results. Given these limitations, it is anticipated that future longitudinal and experimental studies will shed more comprehensive light on the causal mechanisms underlying the relationships between chronotype, work stress, and eating behaviors. Furthermore, the use of convenience and voluntary sampling may have introduced selection and non-response biases, thereby limiting the representativeness and generalizability of the findings. The cross-sectional design also precludes the establishment of temporal or causal relationships between variables. Additionally, the use of self-report measures may have led to common method and reporting biases, and the relatively limited sample size could restrict the generalizability of the findings to the broader population of academic staff. “Furthermore, multiple statistical comparisons were performed without adjustment, which may have increased the possibility of type I error; therefore, the findings should be interpreted cautiously.”

### 4.2 Future directions

Technological advancements, changing educational standards, and the increasing number of universities and students, along with the multifaceted responsibilities of academics—such as scientific productivity, training qualified individuals, and professional development—are increasingly increasing the stress burden in the workplace. It is known that the physiological and psychological changes caused by stress negatively affect academics’ life satisfaction, work performance, and overall health, particularly in terms of nutritional habits, food intake, and body composition. Therefore, developing evidence-based training and support programs to strengthen academics’ capacity to cope with stress is considered a priority for protecting individual health and maintaining institutional productivity. The findings of the current study reveal that work stress decreases with age. Accordingly, equipping young academics with stress management skills, increasing their decision-making autonomy, and strengthening workplace social support mechanisms are critically important for improving both performance and health levels. Furthermore, it is necessary to address the individual, family, and environmental factors that shape work stress from a holistic perspective. Longitudinal and prospective follow-up studies with large and representative samples are needed to gain a more comprehensive understanding of the effects of work stress on eating behaviors and body mass index. In addition, it is considered that follow-up laboratory-based studies that will examine other influencing variables related to work stress in a controlled manner will make significant contributions to the field.

## 5 Conclusion

Academics may experience demanding working conditions accompanied by high levels of work-related stress, fatigue, and challenges related to dietary and sleep behaviors. The findings of the present study indicate that early-career academics had significantly higher levels of work-related stress and fatigue. Work-related stress was also significantly higher among research assistants than among professors and associate professors, suggesting that work-related stress may vary according to academic position and career stage. The observed negative correlations indicate that higher stress levels were associated with lower levels of mindful eating, eating control, and conscious nutrition, while higher fatigue was significantly associated with higher work-related stress. Mindful eating awareness was higher among academics who reported coffee consumption, whereas higher stress levels were observed among those who did not consume coffee. In addition, female academics had higher stress and fatigue levels, whereas male academics reported higher emotional eating tendencies, indicating differences in the behavioral patterns associated with these variables. Overall, the findings suggest that work-related stress, fatigue, and eating-related behaviors are interrelated among academic staff. Based on these findings, university administrations may consider multidimensional support strategies, including chronotype-informed lifestyle planning, mindful eating and eating awareness programs, sleep hygiene support, and workload management. However, given the cross-sectional design of the study, these findings should be interpreted as associations rather than causal relationships. Future longitudinal and intervention-based studies are needed to clarify the temporal and potential causal relationships among work-related stress, chronotype, fatigue, and mindful eating and to inform evidence-based support programs for academic staff.

## Data Availability

The original contributions presented in the study are included in the article/supplementary material, further inquiries can be directed to the corresponding author.
